# Granulomatous Thyroiditis: A Retrospective Analysis of 45 Cases with Histopathological Correlation

**DOI:** 10.5152/eurasianjmed.2026.261553

**Published:** 2026-10-20

**Authors:** Aylin Ege Gül, Sibel Şensu, Ozan Arif Zelyurt, Merve Ak, Sevinç Hallaç Keser, Selma Şengiz Erhan, Nagehan Özdemir Barışık

**Affiliations:** Pathology Clinic, Kartal Dr. Lütfi Kırdar City Hospital, İstanbul, Türkiye

**Keywords:** Follicular nodular disease, granulomatous thyroiditis, papillary microcarcinoma, papillary thyroid carcinoma, subacute thyroiditis

## Abstract

**Background:**

Granulomatous thyroiditis (GT) is a mostly self-limiting inflammatory disorder with an incompletely elucidated etiopathogenesis. This study evaluated the clinicopathological characteristics of GT cases, identified coexisting benign and malignant lesions, and discussed the findings in the context of the current literature.

**Methods:**

A retrospective review of thyroid resection specimens diagnosed with GT was conducted between January 2010 and May 2025. Patient demographics, lesion localization, surgical extent, and concurrent pathologies were analyzed. Logistic regression was used to identify factors predicting malignancy.

**Results:**

A total of 45 cases were included in the study. The mean age of the patients was 50.4 years, and the female-to-male ratio was 3.1:1. Bilateral involvement was detected in 51.1% of cases, and 80% of patients underwent total thyroidectomy. Papillary thyroid carcinoma or papillary microcarcinoma was present in 33.3% of the cases, while benign lesions were observed in 71.1%. Follicular nodular disease was the most frequent benign lesion. In the logistic regression analysis, age, sex, laterality, and surgical extent were not independent predictors of the presence of accompanying malignancy.

**Conclusion:**

Approximately one-third of the GT cases coexisted with papillary carcinoma and two-thirds with follicular nodular disease. Although the high rates might be due to the surgical selection of the cases and cannot be generalized to all cases with GT, larger series are needed for a better understanding of the clinical and pathogenetic aspects of this association.

**Main Points**

- During the 15-year study period, 52 cases of granulomatous thyroiditis were identified which is a relatively large sample size spanning a broad period.
- Approximately one-third of the cases were accompanied by papillary thyroid carcinoma or microcarcinoma. However, the high malignancy rate likely reflects surgical cohort prevalence and cannot be generalized to all cases.
- Follicular nodular disease was the most common benign pathology accompanying GT, observed in approximately two-thirds of cases.

## Introduction

Granulomatous thyroiditis (GT) (subacute thyroiditis, de Quervain thyroiditis) is a non-suppurative, transient, and mostly self-limiting inflammatory disorder of the thyroid gland. The etiopathogenesis and factors influencing its clinical course remain incompletely elucidated.^1^ It most commonly affects individuals aged 30-50 years and is 3-5 times more prevalent in women than men, with an overall reported incidence of approximately 12 per 100 000 population. Patients typically present with fever, neck pain, and thyrotoxic symptoms, and the disease course is characterized by 3 phases: hyperthyroidism, hypothyroidism, and euthyroidism.^1^^-^^6^

Viral infections and genetic predisposition are considered significant contributors to the pathogenesis of GT. A strong association with the *HLA-B35* allele is among the most consistent findings supporting the immunological basis of the disease. Viral antigen presentation via *HLA-B35* molecules is proposed to enhance cytotoxic T lymphocyte activation, leading to destructive granulomatous inflammation in thyroid tissue.^7^^,^^8^ However, the relationship between these immunological mechanisms and coexisting neoplastic or hyperplastic thyroid lesions remains unclear.

Granulomatous thyroiditis is usually diagnosed based on clinical and laboratory findings and managed medically.^3^^,^^9^^,^^10^ Surgical intervention may be required in cases with coexisting nodular pathology, suspicion of malignancy, or clinicoradiological discordance. In such patients, the diagnosis of GT is often established during histopathological examination, which may also reveal concurrent benign or malignant lesions. This underscores the clinical relevance of the association between GT and thyroid neoplasms.

The coexistence of GT with papillary thyroid carcinoma has largely been reported in rare cases or small series, and the prevalence, clinical significance, and potential pathogenetic relationship in larger patient cohorts remain insufficiently studied.^1^^,^^11^^-^^15^ Considering that small and clinically silent neoplastic foci, such as papillary microcarcinomas, may be more frequently detected in surgical series with detailed histopathological examination, reevaluation of the association between GT and malignancy is warranted.

The aim of this study was to evaluate the clinicopathological characteristics of patients diagnosed with GT in the authors’ institution, identify associated benign and malignant lesions, and discuss the findings in the context of current literature. 

## Material and Methods

This retrospective study reviewed cases of GT from thyroid resection specimens archived in the Department of Pathology between January 2010 and May 2025. Hematoxylin–eosin (H&E) stained slides, paraffin blocks, and pathology reports were obtained from the archives. Clinical data were extracted from the hospital information system (HIS). Data collection was conducted meticulously via cross-verification of HIS and physical pathology archive records. To prevent missing cases, all resection specimens from January 2010 to May 2025 were independently screened by 2 researchers.

Clinical evaluation included patient age, sex, lesion localization, type of surgery, and associated benign or malignant pathologies. Histopathological evaluation was performed by 2 experienced pathologists, and discrepancies were resolved through joint review. In cases deemed necessary, serial sections from paraffin blocks were prepared to assess the extent of granulomatous inflammation and avoid missing coexisting microcarcinomas.

The primary objective was to define the morphological spectrum of GT and identify associated neoplastic lesions in surgical specimens. Due to incomplete availability of certain clinical data (e.g., laboratory results), analyses were predominantly based on histopathological findings.

Inclusion criteria were cases with a pathology report indicating “granulomatous thyroiditis,” “subacute thyroiditis,” or “de Quervain thyroiditis,” and availability of suitable paraffin blocks and/or H&E slides. Exclusion criteria included evidence of “tuberculosis, sarcoidosis, fungal infection, foreign body granuloma,” “palpation thyroiditis,” presence of necrosis in granulomas, or absence of suitable material for histopathological evaluation. “True” subacute GT (de Quervain’s) was diagnosed when widespread, multi-follicular, or diffuse parenchymal involvement characterized by the destruction of thyroid follicles, neutrophilic microabscesses were observed. Well-formed granulomas with multinucleated giant cells engulfing colloid were seen in the later stages, accompanied by extensive interfollicular fibrosis. All the patients had pre-operative fine needle aspiration biopsy (FNAB). Fine-needle aspiration biopsy-site changes were focal, restricted, and linear, often showing localized granulation tissue, hemorrhage, hemosiderin pigment, microinfarction, and foreign-body giant cell reactions confined to the needle track which were histologically differentiated from GT which demonstrated diffuse or multi-follicular parenchymal involvement, widespread destruction of thyroid follicles, neutrophilic microabscesses, and well-formed granulomas accompanied by extensive background fibrosis. Palpation thyroiditis cases exhibited strictly focal, limited, and multi-follicular involvement (involving only a few isolated or small clusters of follicles) rather than the widespread, diffuse, or multi-lobular parenchymal involvement that characterizes true subacute GT. In the excluded cases, the histiocytic and multinucleated giant cell reactions were strictly confined insid*e* the follicular lumens, often associated with characteristic clear, macro-vesicular desquamation spaces. In contrast, true GT cases demonstrated complete destruction of the follicular basement membranes with granulomatous inflammation spilling extensively into the interfollicular stroma.

Statistical analyses were conducted using JASP software (version 0.19.4; JASP Team, University of Amsterdam, Amsterdam, The Netherlands, 2024). Continuous variables are expressed as mean ± SD or median (range), and categorical variables as count and percentage. Chi-square or Fisher’s exact test was used for categorical variable comparisons, and Student’s *t*-test or Mann–Whitney *U-*test for continuous variables. Logistic regression analysis was performed to evaluate factors predicting coexisting malignancy, including age, sex, laterality, and type of surgery. A *P*-value <.05 was considered statistically significant.

This study used Google Gemini to improve language and edit grammar during the preparation of the manuscript. However, all content of the study has been reviewed and edited by the authors following the use of AI/LLM tools.

The study protocol was approved by the Kartal Dr.Lütfi Kırdar City Hospital Ethics Committee (Decision No: 2025/010.99/19/27) on August 27, 2025. Patient consent was waived due to the retrospective design of the study, and all patient data were anonymized and de-identified prior to analysis.

## Results

During the 15-year study period, 52 cases of GT were identified in the authors’ pathology archive. Four cases with necrotizing granulomatous inflammation due to tuberculosis, 2 with palpation thyroiditis, and 1 with sarcoidosis were excluded; thus, 45 cases were included (Figures 1-2). The mean age was 50.4 years (range 24-77), comprising 34 women (75.6%) and 11 men (24.4%). Nine patients (20%) underwent lobectomy, and 36 (80%) underwent total thyroidectomy. Bilateral involvement was observed in 23 cases (51.1%), and unilateral in 22 cases (48.9%), with right-sided lesions in 13 (59%) and left-sided lesions in 9 (41%).

Malignancy was present in 15 cases (15/45, 33.3% [CI: 19.6-47.1]). Papillary thyroid carcinoma was detected in 4 cases (4/15, 26.7%) with tumor sizes ranging from 2 to 7 cm (mean: 3.87 cm), while papillary microcarcinomas were identified in 11 cases (11/15, 73.3%) with sizes ranging from 0.05 to 0.9 cm (mean: 0.29 cm) (Figure 3). The features of true subacute GT were explicitly identified in the background non-neoplastic thyroid parenchyma, entirely separate and distant from the tumor sites or microcarcinoma foci.

Benign lesions were observed in 32 cases (32/45, 71.1%), including follicular nodular disease in 30 cases (30/32, 93.75%) and oncocytic adenoma in 2 cases (2/32, 6.25%) (Figure 4, Table 1).

Age, sex, bilateral involvement, and type of surgery were not found to independently predict the presence of malignancy. However, there was a trend toward association with bilateral involvement that did not reach statistical significance (Table 2). Logistic regression lacks adequate power due to the small number of events (15 malignancy cases for 4 variables).

## Discussion

Granulomatous thyroiditis is an inflammatory thyroid disease, often post-viral in origin, with suspected genetic predisposition. While the precise pathogenic mechanisms remain unclear, strong evidence implicates immunogenetic factors, notably the association with *HLA-B35*. Approximately 70% of GT patients are *HLA-B35* positive, supporting an immunogenetic basis.^1^^,^^4^^,^^7^ Viral infections may trigger enhanced antigen presentation via *HLA-B35*, stimulating cytotoxic T lymphocyte activation and destructive granulomatous inflammation. Additional associations with *HLA-B18:01*, *DRB101*, and C*04:01 alleles have been reported, and in Japanese patients, *HLA-Bw67* and *HLA-Cw3* were identified.^1^^,^^4^^,^^7^ Although these data suggest that cellular immune response plays a central role in the pathogenesis of the disease, the occurrence of GT in individuals without any genetic predisposition has slowed down research in this field. The fact that HLA typing was not performed in this study stands as a limitation, restricting the direct evaluation of the relationship between genetic predisposition and clinicopathological features.

Viral infections 2-6 weeks before disease onset are recognized triggers. It is well-established that GT may be of viral or post-viral origin.^1^^-^^4^ Associated viruses include Coxsackie, enteric cytopathic human orphan (ECHO) virus, adenoviruses, influenza, mumps, rubella, parvovirus B19, orthomyxoviruses, HIV, Epstein–Barr virus, hepatitis E, measles, and rarely dengue virüs.^1^^,^^15^ The COVID-19 pandemic highlighted the relationship between Severe Acute Respiratory Syndrome Coronavirus 2 (SARS-CoV-2) infection and GT, with cases reported following both infection and vaccination.^1^^-^^3^^,^^16^^,^^17^ The prevalence of abnormal thyroid functions in individuals who have had COVID-19 has been reported to be approximately 15%; it has been suggested that SARS-CoV-2 may trigger the development of fibrosis by causing damage to thyroid follicular cells, leading to destructive thyroiditis.^18^^,^^19^ Studies on COVID-19 vaccines drew attention to a potential relationship between COVID-19 vaccination and thyroid dysfunction, particularly thyroiditis; among the thyroid issues emerging post-vaccination, GT (60%) and Graves’ disease (25%) were the most frequently observed.^16^ A notable limitation of this study is the insufficient data on recent viral infections or vaccination status, which prevents a more thorough analysis of the triggering factors for GT development in this study’s cohort.

In addition to these proposed etiological factors, treatments that modulate the immune system have also been associated with the development of GT.^1^^,^^20^^-^^23^ More recently, the gut microbiota–thyroid axis has emerged as another potential mechanism involved in the etiopathogenesis of GT.^24^

The female-to-male ratio of 3:1:1 in this series is consistent with the literature, with female patients being predominant as expected.^1^^-^^4^ The observation of bilateral involvement in the majority of cases can be explained by the fact that the disease, initially unilateral, may spread to the contralateral lobe over time. The high rate of total thyroidectomy in this study can be attributed to the fact that the research was conducted on surgical specimens and that GT is often diagnosed histopathologically in patients operated on due to accompanying nodular pathologies or suspicion of malignancy. This situation may lead to a selection bias, suggesting that this series may not represent all GT cases.

One of the most remarkable findings of this study is the coexistence of papillary thyroid carcinoma (PTC) or papillary microcarcinoma in approximately one-third of the GT cases. In the literature, the association between GT and papillary carcinoma is rarely reported, typically appearing in the form of case reports.^1^^,^^11^^,^^12^^,^^14^^,^^15^ Existing studies have reported the coexistence rate of PTC and GT to be around 3-4%.^1^^,^^4^^,^^14^ In the authors’ opinion, one of the reasons for the high coexistence rate in the series is the high prevalence of papillary thyroid carcinoma in the authors’ country. Furthermore, the fact that the authors’ series was based on surgical specimens and that all specimens were subjected to detailed histopathological examination may have increased the likelihood of detecting small, clinically silent foci, such as papillary microcarcinomas. This suggests that the relationship between GT and papillary thyroid carcinoma may be related to increased diagnostic sensitivity and sampling density rather than a direct causal link. However, whether this association is related to the effects of inflammation on the tumor microenvironment or is merely an incidental finding cannot be clearly established with the design of this study.

In the logistic regression analysis conducted to evaluate factors predicting the presence of malignancy, age, sex, laterality, and type of operation were not identified as independent predictors. However, a borderline trend toward a relationship was observed for bilateral involvement, although it did not reach statistical significance. While this finding suggests potential effects of widespread inflammation on the detectability of accompanying neoplastic foci, it should be interpreted with caution due to the limited sample size.

When evaluated in terms of coexisting benign lesions, follicular nodular disease was most frequently observed in this series. Large series examining the association between GT and benign thyroid pathologies are limited in the literature; in this regard, this study provides a significant descriptive contribution to the field.

It has been reported that the clinical characteristics of the disease have been changing in recent years, with a notable observation that neck pain and fever do not accompany all cases.^1^^,^^3^^,^^4^^,^^6^ Some cases present with non-specific symptoms such as fatigue, fever, and palpitations. During the transient hyperthyroid phase, if thyroid hormone levels are elevated, more common conditions such as Graves’ disease or toxic multinodular goiter are often initially considered. As the disease progresses, the transition from hyperthyroidism to hypothyroidism can lead to additional diagnostic challenges.^5^ Until recently, the absence of thyroid antibodies was considered a characteristic finding in GT; however, it is increasingly reported that a significant number of cases show elevations in antithyroid antibodies, such as thyroid peroxidase antibodies, thyroglobulin antibodies (TgAbs), and thyrotropin receptor antibodies.^1^ In some patients, the disappearance of TgAb within 4 months suggests that the emergence of these antibodies may be a consequence of thyroid antigen release due to glandular damage.^1^ In these patients, it is also necessary to investigate whether there is an increased coexistence with autoimmune thyroid diseases.

The treatment of GT is symptom oriented; while non-steroidal anti-inflammatory drugs (NSAIDs) are sufficient for mild cases, corticosteroids are preferred for moderate-to-severe cases.^3^^,^^9^^,^^10^ In an analysis prepared with pooled data, the recurrence rate was reported as 12%. According to the analysis, the risk of recurrence is higher in the glucocorticoid-using group compared to the NSAID-using group (Relative Risk = 1.84, 95% CI = 1.04-3.24). The authors concluded that further randomized controlled trials and prospective cohort studies, as well as investigations to elucidate molecular and cellular mechanisms, are required to predict recurring vs. non-recurring groups, thereby preventing early recurrence and ensuring appropriate treatment.^10^

The primary limitations of the study include its retrospective design and the potential selection bias arising from the reliance on surgical specimens. The fact that a large proportion of cases in this series underwent surgery due to nodular pathologies or suspicion of malignancy may have resulted in a higher malignancy rate compared to the general population with GT. A very high (33%) malignancy rate is probably related to surgical cohort prevalence and should not be considered representative of the general GT population. Also, the logistic regression used only 15 malignancy cases for 4 variables, which is too small for reliable results, which can be considered a limitation. Additionally, the inability to systematically access patients’ inflammatory markers at the time of presentation (e.g., erythrocyte sedimentation rate, CRP) and detailed clinical histories (such as infection etiology, vaccination status, and immunomodulatory treatment data) limited the authors’ ability to perform a thorough clinicopathological correlation

Conversely, this study is valuable for its relatively large sample size from a single center, spanning a broad 15-year period. The independent and detailed histopathological examination of all specimens by 2 experienced pathologists during the data collection and analysis process ensured high diagnostic sensitivity, particularly in detecting clinically silent foci such as papillary microcarcinoma. This approach enhanced the methodological strength of the study and provided the literature with a significant dataset regarding the coexistence of GT and neoplastic processes.

In conclusion, it was found that approximately one-third of the GT cases were accompanied by papillary thyroid carcinoma, and two-thirds were associated with follicular nodular disease. Studies involving larger patient series are required to better understand this association from both clinical and pathogenetic perspectives. Furthermore, while the response to medical treatment in GT is generally high, considering that recurrence can occur in approximately 12% of cases, identifying subgroups at high risk for recurrence will contribute to the optimization of treatment and follow-up strategies.

## Figures and Tables

**Figure 1.**
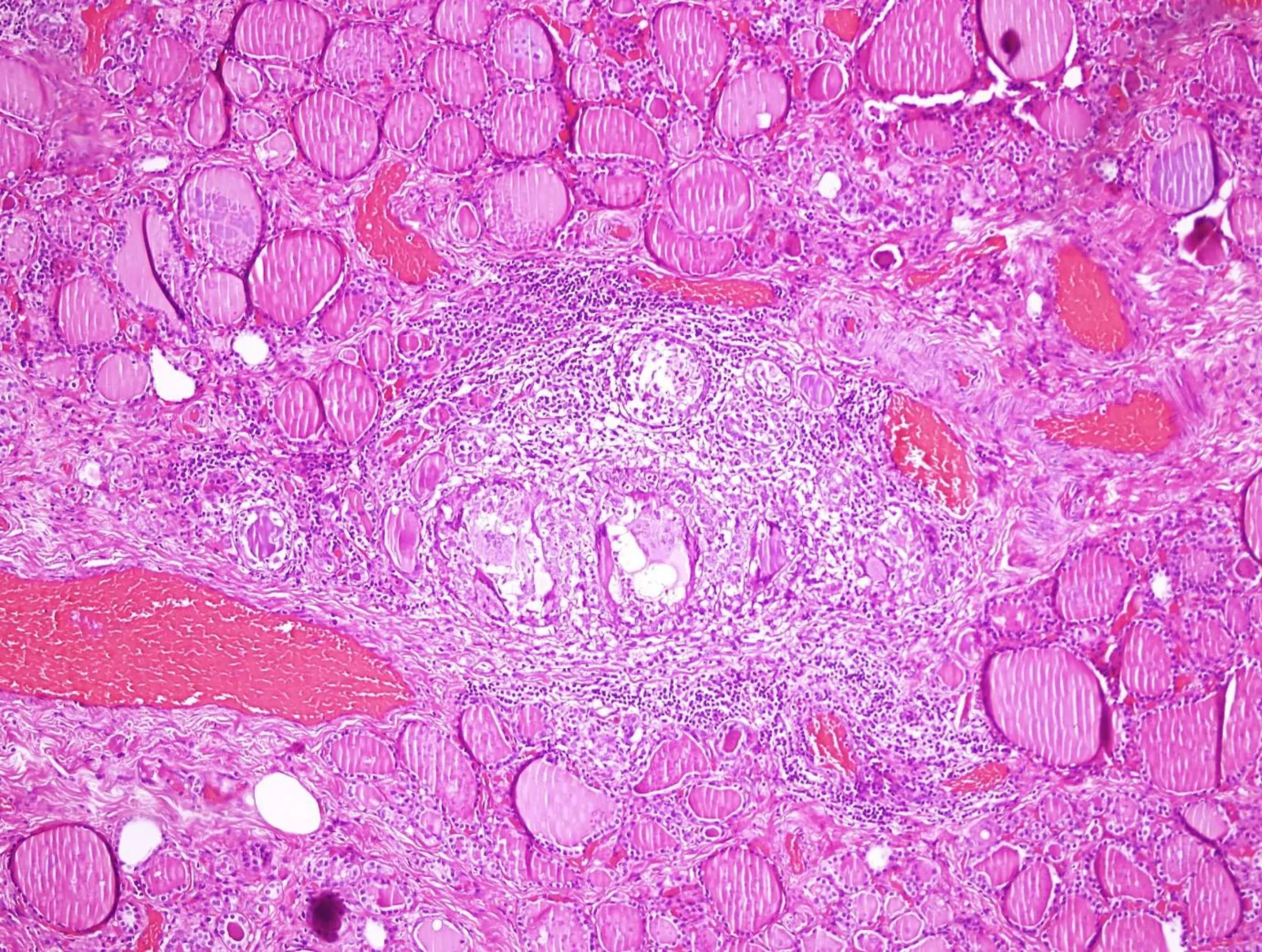
Granulomatous thyroiditis showing granuloma formation (H&E ×100).

**Figure 2.**
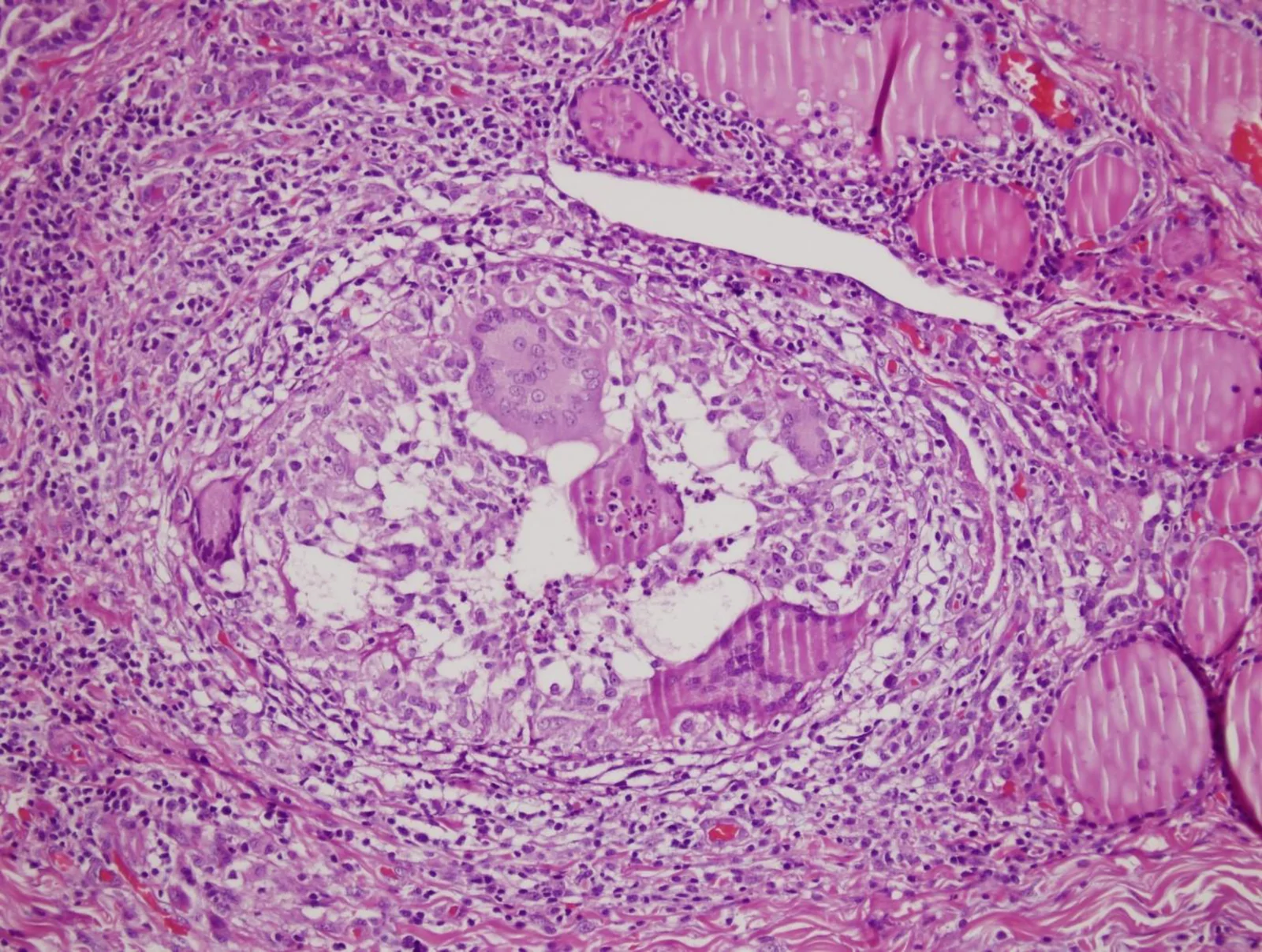
Granuloma with multinucleated giant cells and neutrophils (H&E ×200).

**Figure 3.**
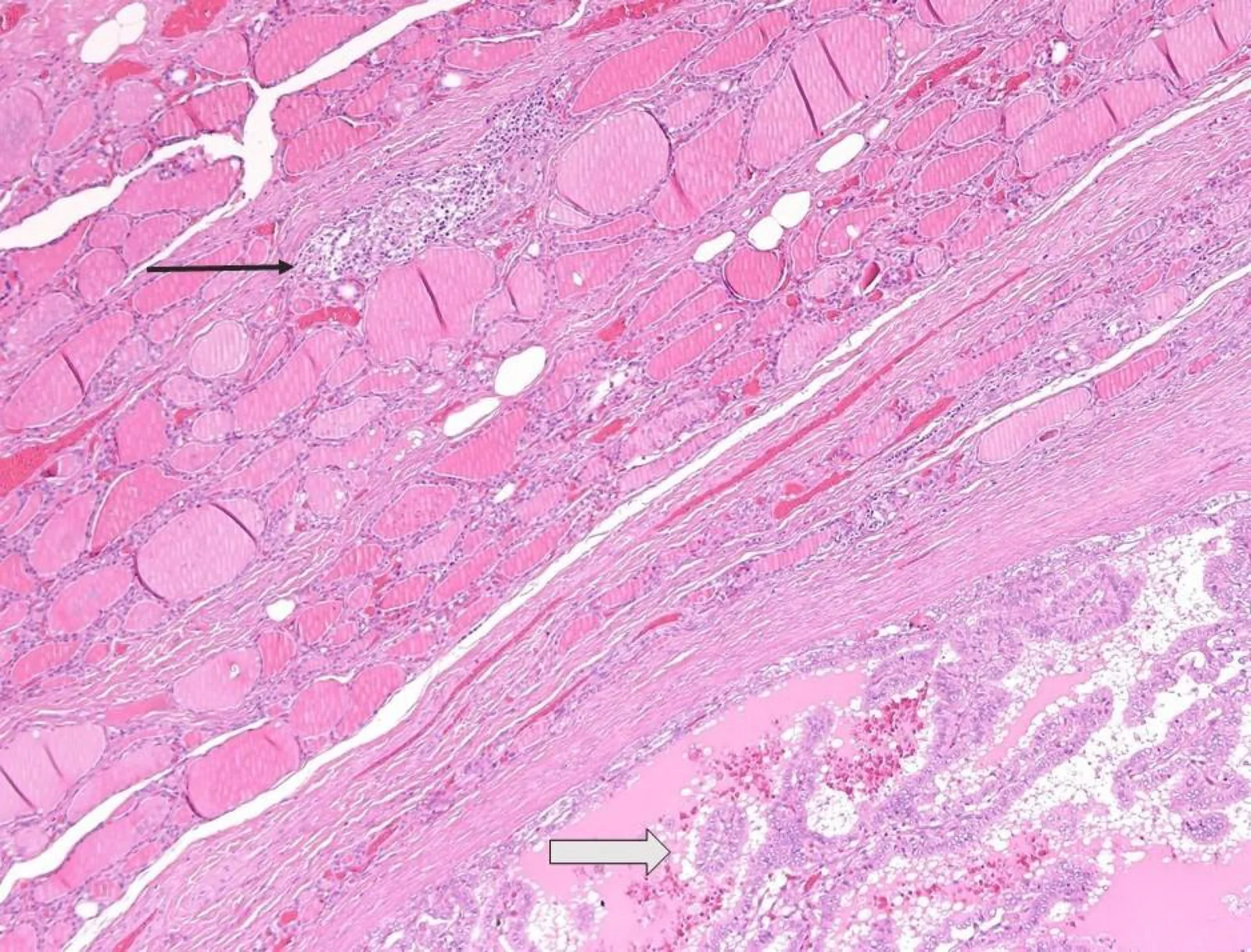
Coexistence of granulomatous thyroiditis (black arrow) and papillary thyroid carcinoma (white arrow) (H&E, ×100).

**Figure 4.**
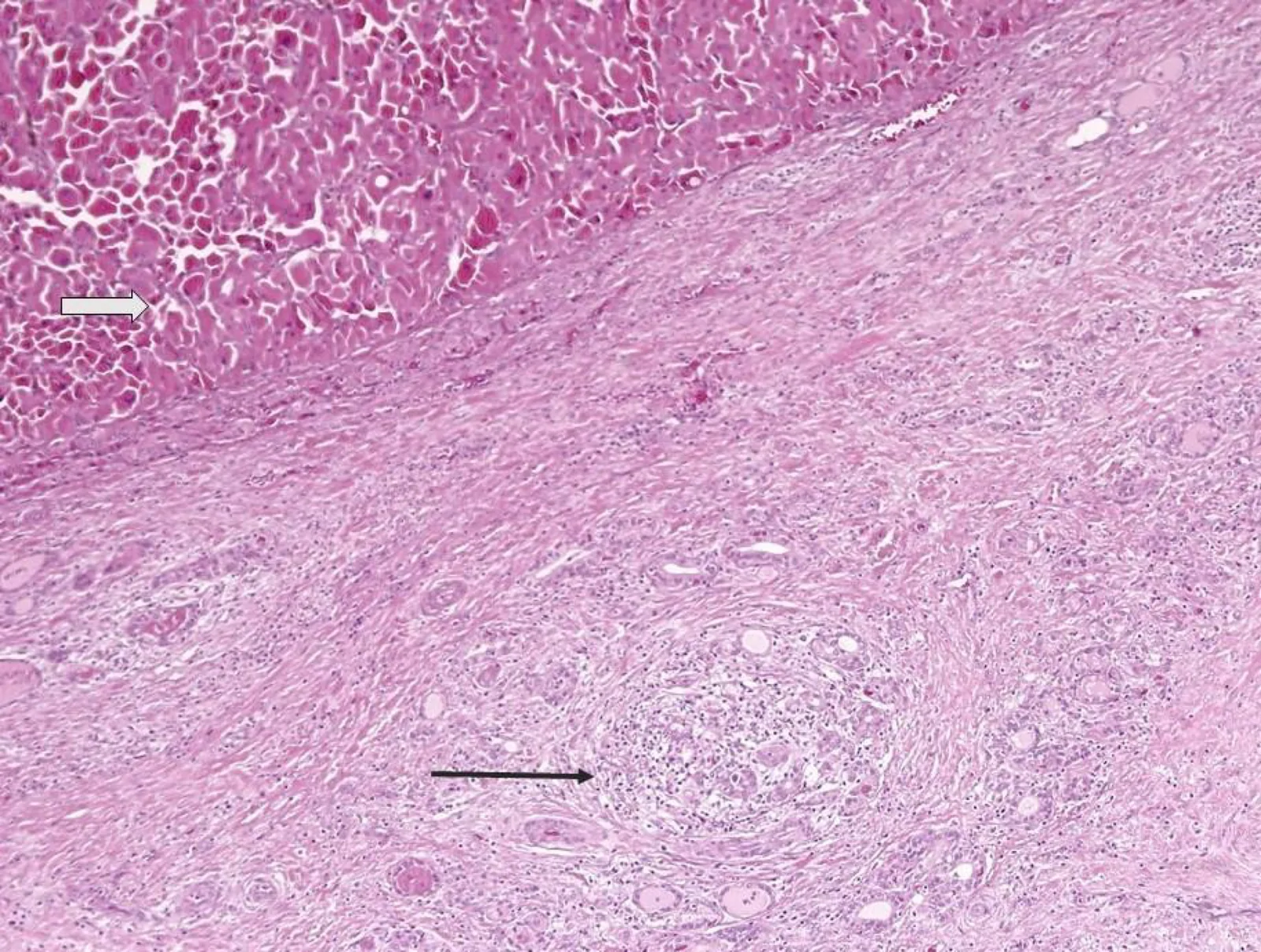
Granulomatous thyroiditis (black arrow) adjacent to an oncocytic adenoma (white arrow) (H&E, ×100).

**Table 1.** Clinicopathological Characteristics of the Cases (N = 45)

Parameter	Number (n)	%
Gender		
Female	34	75.55
Male	11	24.44
Laterality		
Bilateral	23	51.11
Unilateral	22	48.88
Right	13	59.09*
Left	9	40.9*
Surgical extent		
Lobectomy	9	20
Total thyroidectomy	36	80
Coexisting malign lesions (n = 15)		
Papillary thyroid carcinoma	4	26.66
Tumor size	3.87 cm (2-7 cm)	
Papillary microcarcinoma	11	73.33
Tumor size	0.29 cm (0.05-0.9 cm)	
Coexisting benign lesions (n = 32)		
Follicular nodular disease	30	93.75
Oncocytic adenoma	2	6.25

The percentages for malignant and benign lesions were calculated within their respective subgroups.

*Percentages were calculated based on unilateral cases.

**Table 2.** Logistic Regression Analysis of Factors Predicting Malignancy in Granulomatous Thyroiditis

Variable	OR	95% CI	*P*
Age	0.97	0.92-1.03	.323
Gender (male)	0.34	0.05-2.17	.253
Bilateral involvement	0.21	0.04-1.01	.052
Total thyroidectomy	2.86	0.44-18.47	.271

OR, odds ratio.

## Data Availability

The data that support the findings of this study are available on request from the corresponding author.
